# The Festal Number of *Hygiea*1Hygiea; Medicinsk och Farmacevtisk Manadsskrift, utgifven af Svenska Läkare Sällskapet; Festband, med Anledning af Tidskriftens Femtioäriga Tillvaro, med. 6 Taflor och 28 Afbilnin, gar i Texten, Stockholm: Samson & Wallin. 1889.

**Published:** 1890-01

**Authors:** 


BUFFALO MEDICAL AND SURGICAL JOURNAL

A MONTHLY REVIEW OF MEDICINE AND SURGERY.

*EDITORS:*

THOS. LOTHROP, M. D. - - - W. W. POTTER, M. D.

All communications, whether of a literary or business character, should be addressed to the editors: 284 FRANKLIN STREET, BUFFALO, N. Y.

### Editorial.

### THE FESTAL NUMBER OF HYGIEA.[1]

THE ever-welcome *Hygiea* has just made its appearance in a most remarkable and charming form. It is not, however, the regular monthly number, being in fact a volume of nearly 500 pages. As above, the title-page informs us that it is a "Festal number in reference to the existence of the periodical for fifty years, with six engravings and twenty-eight illustrations in the text."

For beauty of letter-press nothing can be superior, and a glance at its contents shows that no pains have been spared to make the book—for such in fact it is—a valuable addition to a physician's library. It contains in all eighteen contributions by the most learned physicians and chemists of Sweden, several of which are exhaustive monographs on the subjects of which they treat. They are written in various languages evidently with the purpose of reaching those who are unacquainted with the mother-tongue of the writers. We may here, in passing, explain that the Swedish student of medicine is compelled to acquire a fair knowledge of three modern languages at least before he can finish his professional studies, since important scientific works by English, French, and German writers are seldom translated into his own vernacular.

The group of portraits, which faces the title page, are those who have been the editors-in-chief since the first year of the life of the periodical (1839), and it is difficult to find nobler and more intelligent heads anywhere.

The first article is written by Martin Sondén, and is, of course, historical. A brief but comprehensive glance is cast over the history of the medical literature of the Scandinavian nations, particularly that of Sweden, up to the time of the establishment of *Hygiea*. The first

1. Hygiea; Medicinsk och Farmacevtisk Manadsskrift, utgifven af Svenska Läkare Sällskapet; Festband, med Anledning af Tidskriftens Femtioäriga Tillvaro, med. 6 Taflor och 28 Afbilnin, gar i Texten, Stockholm: Samson & Wallin. 1889.

attempts to found periodicals in the service of the healing art took place simultaneously in Stockholm and Gothenburg in 1781. The latter did not survive its first year, while that of the former continued, under various names and vicissitudes, to exist till 1807, when it too expired. In 1783 appeared in Stockholm the first volume ("Tomus Primus") of Acta Medicorum Svecicorum, a somewhat voluminous work containing fifteen articles in Latin. The second volume of this was never written. Many other attempts at periodical medical literature were made, but though some of these were for the time able productions, no actual stability was attained till the foundation of the *Hygiea* in 1839 by members of the Swedish Society of Physicians, whose organ, entitled "Transactions of the Society of Swedish Physicians," was incorporated in it, and has since constituted a part of the publication. Since the establishment of *Hygiea* a large number of periodicals on various subjects more or less related to medicine have made their appearance, some of which have had but a brief existence, while others are still doing excellent work, being most ably conducted.

Among the defunct may be mentioned Dr. Lagberg's *Friend of Health*, a publication which seems to have anticipated the mind-cure. It undertook to show that cold water mingled with spirit (breath, not whiskey) would drive away syphilis, rheumatism, gout, etc., etc., from the body—and yet even Dr. Lagberg had his diploma.

Neither did homeopathy take a scientific root in Sweden, since the only attempt to establish an organ in its interest by one Dr. Liedbeck, who appears to have been something of a crank, began and ended in one year. Its title was, to say the least, most extraordinary: *Instruction in Homeopathy for the Swedish People; Directions for Health and Care of the Sick, Nature-healing and Self-help, Without and With Medicaments in Diseases among Men and Cattle.* The author further remarks: "Liedbeck, a versatile man, with a strong inclination to the *bizarre* as well in word as thought, still believed in his homeopathy. Since his day we certainly have heard homeopathy talked of in our country, but fortunately not within the medical world. It has now-a days become a means of living for spinsters, quacks, unfortunate affairs-men, and other charlatans who live upon the credulity and simplicity of the masses."

In citing these remarks of the author we disclaim any intention to cast odium on the well-informed disciples of Hahnemann of our country, most of whom, fortunately for their patients, practice their profession in a manner little resembling the teachings of their reputed master, our sole purpose being to point out the slight esteem in which his doctrines are held by European scientists.

Among the successful periodicals which are issued in the Scandinavian countries in the interest of medical literature may be mentioned the *Nordisk Medicinskt Archiv*, a quarterly common to Sweden, Norway, Denmark, and Finland, a most ably conducted production, and one of our own valued exchanges. Its editor-in-chief is Dr. Axel Key, Professor of Pathology and Anatomy in Stockholm. His coadjutors are among the ablest contributors to medical literature in the principal seats of learning where the Scandinavian languages are spoken. Three of these live in Helsingfors, Finland; three in Christiania, Norway; three in Copenhagen, Denmark; and the rest are Swedes from Upsala, Lund, and Stockholm. Several other monthly, bi-monthly, and quarterly publications still have a lively existence in Sweden, though a large number of greater or less consequence have expired, but those that are living rest on a true scientific base.

Dr. Sonden closes his sketch with the following words:

The notices we have contributed only touch upon the external outlines of the development of medicine in our country so far as these may be marked by periodical literature. But, however evanescent, faulty and imperfect these notices may be, they still show that *Hygiea*, which made its appearance just at the dawn of a new era, also was an expression of the want of this new era. They also point out that the interval, during which *Hygiea* has lived and worked, in a greater degree than formerly, has been distinguished by earnest work and a many-sided powerful development. Who will deny that this augurs good for the future of the medical science of Sweden?

Numbers two and three are written in German. Professor Gustaf Retzius in the former discourses on the formation of the ovaria and the Græafian Follicles, describing his investigations on live rabbits and illustrating the results in finely executed lithographs. In the latter, Dr. E. J. Widmark gives an interesting account of the Influence of Light on the Skin, showing that its action in producing diseased conditions of the integument, such as eczema solaries, etc., is due to the ultra violet rays, and that the same condition may obtain by a near exposure to electrical light. These theories are supported by reference to many well-known historic instances, as well as by actual experiments on lower animals, and appear to be well sustained.

In the fourth number Dr. J. G. Edgren describes his studies on Pulsus Bigeminus. A rapid glance at the contents gives assurance that the author is master of his subject; especially are the cases adduced in illustration of absorbing interest. The beautiful cardiographical and sphygmographical delineations, which are enlarged by the pantagraph and printed in white lines on black, are exceedingly curious, and cannot fail to be suggestive to the pathological student, even though unable to read the Swedish text.

The fifth paper is written in German by J. E. Johanson and Robert Tigerstedt, of the Carolinian Medico-Chirurgical Institute at Stockholm.

A series of careful observations and experiments on the action of the pneumogastric nerve upon the movements of the heart are here exhibited. The subject has been studied *de vivo* by vivisection, and the results recorded by registering instruments and printed in the text.

The sixth article is in Swedish by Severin Jolin, and treats of the effect produced upon the alkaline excretions of carnivorous animals by neutral elements, which within the organism are converted into acids. The experiments which were made on dogs are carefully tabulated.

Quelque cas de polyopie monoculaire hysterique. Memoire de la clinique de maladies du système nerveux à Stockholm, par C. G. Santesson is the title of the next number, which, as indicated, is written in French. The length of this paper precludes the possibility of even the briefest synopsis, but a glance through its sixty-four pages convinces us that it is a most interesting and valuable contribution to medical literature.

In number eight Prof. P. J. Wissing has a communication in German, entitled Zur Kasuistik der Augenmigraine, besonders der mits cerebralen Erscheinungen associirten Form. After a few remarks on the history of this form of malady founded on the literature of preceding writers, especial regard being paid to the opinions of Charcot and Fêrès, the author proceeds to give his latest observations of six cases which have come under his eye at his nerve clinic in Stockholm. The article is fifty-nine pages long, and closes with a brief account of the opinions of the various authorities as to the cause of the disease.

Number nine is a short article in Swedish by Alb. Lindström, describing two scaphocephalous sculls in his possession, illustrated by beautiful outline engravings.

The next contribution by Gustaf Ritzius, also in Swedish, and illustrated with tables and photographs, describes at length Bertillon's anthropometrical method of identifying criminals in thirty-six pages.

Number eleven by E. O. Hultgren and E. Landergren, of the Physiological Laboratory of the Carolinian Medico-Chirurgical Institute at Stockholm, a German article, is entitled Untersuchung die Ernährung bei freie gewählter Kost,—fifty-seven pages, profusely explained by tabulated statements.

Next follows (No. 12) a description by Prof. Carl J. Rossander, of a case of pylorus and of section of intestine with fatal result. (Swedish.)

The author remarks in the opening paragraph that it is much more agreeable for an operator to describe a case with happy issue, but he holds that an unfortunate result of an operation may be no less instructive. The case here cited is one of unusual interest, and may in a translated form find place in one of our issues in the near future.

"A few words on the exhibition and determination of sugar in the urine," by Prof. K. A. H. Mörner, next claims the reader's attention. On looking over it, we find it to be a careful and highly scientific review of all the known methods for determining the presence and quantity of the various kinds of sugar in the human urine, whether in health or sickness, particular care being taken to point out the simplest and best means of investigation by the practising physician—an article that merits careful study.

The Transposition Products of Phenacetin in the human organization is the title of the next paper by the same author.

The chemical changes of various antipyreal and antineuralgical aniline or phenolin remedies are briefly discussed, the poisonous effect on the blood of some of them pointed out, and chemical remedies suggested to render them inocuous.

No. 15. A case of hernia diaphragmatica acquisita, by Dr. E. S. Perman and Prof. C. Wallis. This interesting case must, for want of space, be left to further study and possible translation.

In the sixteenth number Docent F. Westermark and M. G. Annell give an interesting case of glandular myxomatic ovarian cyst, with peculiar myxom-like forms in the cavity of the peritoneum, beautifully illustrated.

The seventeenth article treats of the manner of supplying materials for anatomical studies in Stockholm, Upsala, and Lund, by Prof. Algot Key-Àberg. The next is from the pen of Karl Rudberg, and is an appeal for the re-organization of the medical department of the navy.

Thus we have in the briefest manner sketched the contents of this valuable contribution to medical literature. The scope of our plan in editing our journal does not permit a more extended notice, however much we would be pleased to give to each article the attention it deserves. In calling to mind the grave matters discussed in some—we might well say in all—of the numbers, we cannot but regret that our view was necessarily cursory, and that we have even been unable to give our readers the benefit of extracts of what may be termed a compendium of medical lore, rarely found in one moderately-sized volume, and coming from careful investigators, some of whom enjoy and deserve a world-wide reputation. Besides and apart from the intrinsic value of its pages, they give the greatest proof of the brotherhood of science. The true scientist is your only cosmopolitan. In search of truth he knows no country, no nationality, no jealousy, that will bar him from looking for it in the remotest corner of the earth. Nor will he refuse credit where credit is due. It is gratifying also to know that the jealousies existing between the Scandinavian nations, which to a large extent are fostered by writers of fiction, seems to be utterly unshared by the members of the medical profession, and that from the fiords of Norway, from the fertile fields of Denmark, nay, even from the plains and moors of Finland, knowledge and wisdom finds in Sweden a cordial welcome and due appreciation. May northern lights be ever radiant.

